# Prognostic value of pretreatment neutrophil-to-lymphocyte ratio in renal cell carcinoma: a systematic review and meta-analysis

**DOI:** 10.1186/s12894-020-00665-8

**Published:** 2020-07-06

**Authors:** Yuan Shao, Bo Wu, Wei Jia, Zikuan Zhang, Qian Chen, Dongwen Wang

**Affiliations:** 1 Shanxi Medical University, Taiyuan, 030001 Shanxi People’s Republic of China; 2 Department of Urology, First Hospital of Shanxi Medical University, Taiyuan, 030001 Shanxi People’s Republic of China; 3 National Cancer Center/National Clinical Research Center for Cancer/Cancer Hospital & Shenzhen Hospital, Chinese Academy of Medical Sciences and Peking Union Medical College, Shenzhen, 518116 People’s Republic of China

**Keywords:** Inflammation, Neutrophil-lymphocyte ratio, Biomarker, Kidney neoplasms, Prognosis, Survival, Meta-analysis

## Abstract

**Background:**

Numerous studies show that the pretreatment neutrophil-to-lymphocyte ratio (NLR) is associated with the prognosis of patients with RCC. However, their findings are inconsistent, urging us to explore the prognostic value of NLR in RCC patients.

**Methods:**

This study was pre-registered in PROSPERO (CRD42020167131). Two reviewers independently performed a systematical search of PubMed, Web of Science, EMBASE, and Cochrane Library databases for prospective or retrospective cohort studies investigating the prognostic value of pretreatment NLR. Hazard ratios with 95% confidence intervals for overall survival (OS), disease-free survival (DFS), progression-free survival (PFS), cancer-specific survival (CSS), and other useful clinicopathological features were extracted and analyzed with fixed or random-effect models by using Review Manager 5.3 and Stata 12.0 software. Heterogeneity was estimated on the basis of Cochran’s Q test and *I*^*2*^ value. Sensitivity analyses and subgroup analyses were also performed to explore the potential sources of heterogeneity. Publication bias was assessed with funnel plots and precisely assessed by Egger’s tests. The quality of the evidence was evaluated in accordance with the Grading of Recommendations Assessment, Development, and Evaluation (GRADE).

**Results:**

Overall, 6461 RCC patients from 24 retrospective studies and 1 prospective study were included. In overall population, elevated pretreatment NLR was associated with poorer OS (pooled HR = 1.90, 95% CI = 1.56–2.30, *p* < 0.001; *I*^*2*^ = 87%), DFS/PFS (pooled HR = 2.09, 95% CI: 1.49–2.94, *p* < 0.001; *I*^*2*^ = 99%), and CSS (pooled HR = 2.31, 95% CI: 1.61–3.33, *p* < 0.001; *I*^*2*^ = 14%). Furthermore, this negative association was further confirmed in patients with nonmetastatic and metastatic RCC patients, respectively. We also investigated the predictive role of NLR in metastatic RCC patients treated with immune checkpoint inhibitors (ICIs). The results indicated that the level of NLR was significantly associated with OS (pooled HR = 3.92, 95% CI: 2.00–7.69, *p* < 0.001; *I*^*2*^ = 0%) and PFS (pooled HR = 2.20, 95% CI: 95% CI: 1.61–3.01, *p* < 0.001; *I*^*2*^ = 20%).

**Conclusions:**

This study demonstrated that elevated pretreatment NLR was significantly associated with poor prognosis of RCC patients. NLR could be helpful as a potential prognostic biomarker to guide clinical decision-making and select individualized treatment strategies for RCC patients.

## Background

Renal cell carcinoma (RCC) is a common malignant cancer of the urinary system; its morbidity and mortality have been increasing in recent years [1, 2]. When RCC is identified early by imaging conducted for other reasons serendipitously, long-term survival is generally excellent. When RCC is detected with symptoms, the prognosis is poor [3]. However, reliable biomarkers suitable for clinical application remain undiscovered worldwide, and presently except for imaging examination, no method is effective for the early diagnosis and prognosis of RCC. Therefore, in order to improve the prognosis of RCC patients and guide clinical decision-making, it is necessary to identify reliable pretreatment biomarker to diagnose, monitor, and manage this disease.

There is growing evidence indicating that immune response and systemic inflammation is the crucial component of human cancer development and progression [4–6]. Several studies have indicated that pretreatment neutrophil-to-lymphocyte ratio (NLR), as a systemic inflammatory biomarker, was associated with the prognosis of patients with malignancies; thus providing a new perspective for predicting the prognosis of cancer [7–9]. More recently, various studies evaluated the prognostic value of NLR in RCC patients, whereas their conclusions are controversial [10, 11]. In 2019, a meta-analysis reported that NLR is a predictor associated with prognosis in RCC patients. However, this study did not perform sensitivity analysis and subgroup analysis to explore the potential sources of heterogeneity and assess the publication bias [12]. Therefore, the aim of this systematic review and meta-analysis was to provide a systematical and comprehensive perspective clarifying the prognostic value of pretreatment NLR for both non-metastatic and metastatic RCC patients.

## Methods

### Search strategy

This study was pre-registered in PROSPERO (CRD42020167131) and conducted in accordance with the guidelines of the Preferred Reporting Items for Systematic Review and Meta-Analysis (PRISMA). A comprehensive online literature search was performed to select the potential studies on PubMed, Web of Science, EMBASE and Cochrane Library databases from inception to December 2019. The main terms used in our search strategy included the following: (“renal” or “kidney”) and (“carcinoma” or “neoplasms” or “cancer” or “tumor”) and (“NLR” or “neutrophil-lymphocyte ratio” or “neutrophil-to-lymphocyte ratio”).

### Inclusion and exclusion criteria

The enrolled studies should meet the following inclusion criteria: (1) prospective or retrospective cohort studies evaluating the association between the pretreatment NLR and overall survival (OS), disease-free survival (DFS), progression-free survival (PFS), cancer-specific survival (CSS) of the patients with RCC; (2) patients in these studies did not receive any treatment before obtaining samples; (3) NLR was collected within 30 days before treatment; and (4) the study directly provided hazard ratios (HRs) with 95% confidence intervals (CIs) or had sufficient data to calculate these statistics. If the data in the studies were duplicated, only the data from the most recent study were used. The studies were excluded based on the following exclusion criteria: (1) studies without sufficient survival data for further analysis, (2) duplicated studies or publications, and (3) expert opinions, meeting abstracts, editorials, case reports, letters, reviews or meta-analysis.

### Date extraction

For each eligible study, two separate authors independently extracted the following items: study characteristics (first author’s name, recruitment region, year of publication, type of study, and sample size); patient information (gender, age, and race), pathological characteristics (TNM stage and histology subtype), disease setting (localized or metastatic), NLR cut-off values (the number and/or percentage of patients with high NLR versus those with low NLR), clinical features (treatment strategy, patient’s survival outcome, and follow-up duration), OS, DFS, PFS, and CSS outcomes expressed as HRs (and 95% CI) for RCC patients with high pretreatment NLR versus patients with low pretreatment NLR. In the case of disagreements between individual judgments, the consensus was achieved by discussion with the third investigator.

### Quality assessment

The quality of each enrolled study was assessed using the Newcastle-Ottawa Scale (NOS), which consists of three factors: selection, comparability and exposure [13]. The highest score is 9 points and studies with scores 7 or more, 4–6, and lower than 4 were respectively considered to have a low, moderate, and high risk of bias. Any disagreement was resolved by discussion with the third reviewer.

### Statistical analysis

Coprimary end-points of the present meta-analysis were OS, DFS, PFS, and CSS in all patients and in patients with nonmetastatic or metastatic RCC. When the included studies directly reported the survival analysis, the HRs and 95% CIs were extracted and used to calculate pooled HRs; otherwise, the Engauge Digitizer software (version 4.1) was used to compute and estimate these data from the Kaplan-Meier survival curves [14, 15].

Cochran’s Q test and *I*^*2*^ statistic were used to assess the heterogeneity among the included studies. If significant heterogeneity existed (*I*^*2*^ > 50% and/or *P* < 0.10), the pooled HRs and 95% CIs were calculated by a random-effect model; otherwise, the fixed-effect model was performed (*I*^*2*^ < 50% and/or *P* > 0.10) [16]. Sensitivity analyses were conducted to assess the stability of the results by sequentially omitting a single study at a time. Subgroup analyses were conducted to explore the potential sources of heterogeneity. In addition, funnel plots and Egger’s tests were used to assess the risk of publication bias. Egger’s test and the trim and fill method were performed with Stata 12.0 software (STATA Corporation, College Station, TX, USA). Other statistical analyses were conducted using Review Manager 5.3 software (Cochrane Collaboration, Copenhagen, Denmark). All *p*-values were two-sided, and a statistically significant difference was defined as *p* < 0.05.

### Quality of evidence

The quality of the evidence of the predictive value of pretreatment NLR for the prognosis in RCC patients was assessed according to the Grading of Recommendations Assessment, Development, and Evaluation (GRADE) [17].

## Results

### Included literature

**Fig. 1:**
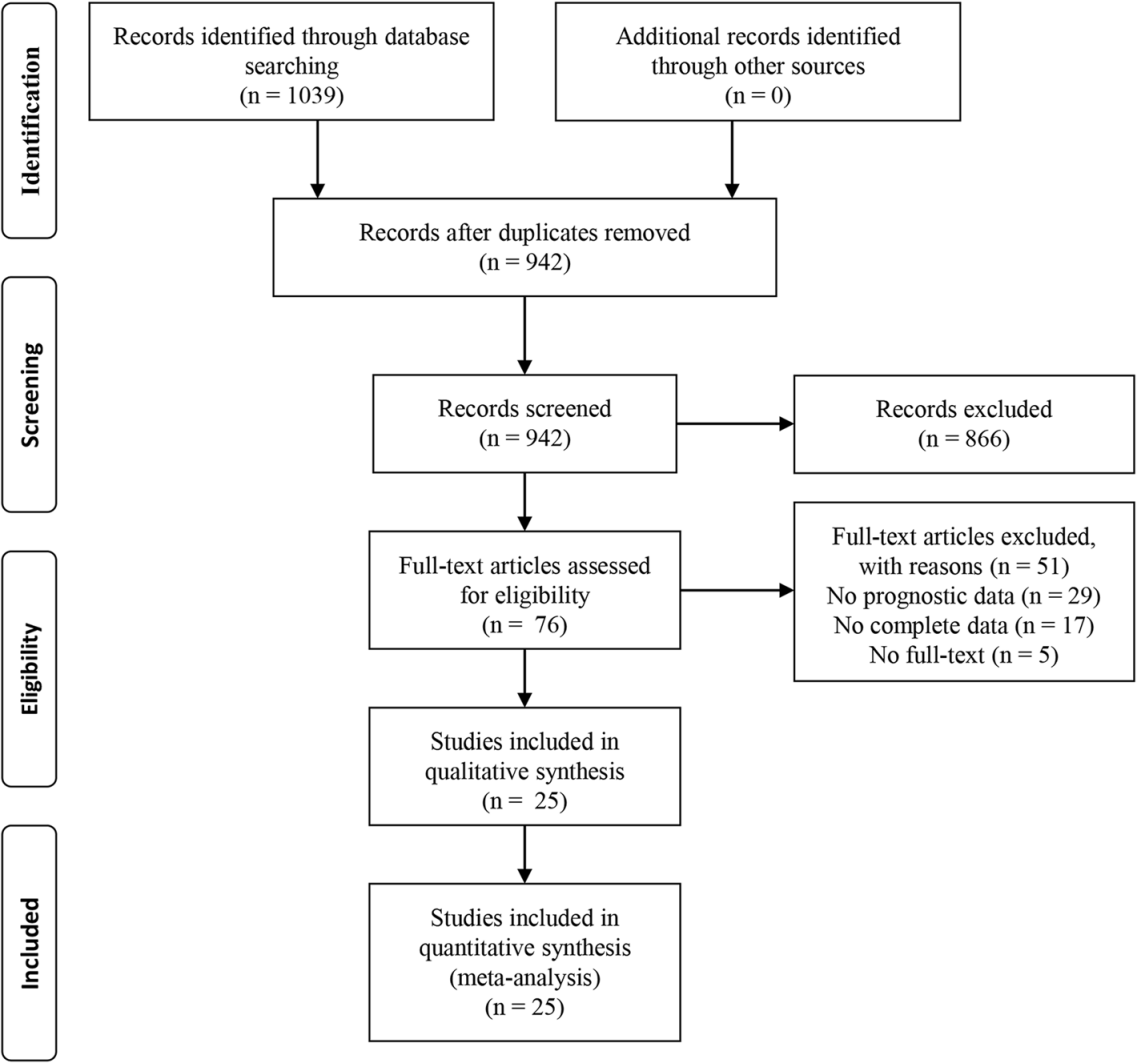
Flowchart of the eligible studies in the current meta-analysis

Based on our search strategies, we identified 1039 potentially relevant studies. After removing duplicates, we viewed the titles and abstracts of the remaining 942 records. Subsequently, we assessed the full text for 76 articles. Finally, 25 studies were enrolled in the present meta-analysis [10, 11, 18–40]. The study selection process is presented as a flowchart in Fig. 1.

### Study characteristics

Overall, 6461 patients with RCC were included. Table 1 showed the main characteristics of the 25 enrolled studies. For the study design, 24 were retrospective cohort studies and published between 2010 and 2019. Twelve studies reported localized/non-metastatic RCC, and 13 reported metastatic RCC. Among the 25 studies, OS was reported in 19 studies, DFS or PFS in 18 studies, and CSS in 4 studies. Histology type includes clear cell RCC, papillary RCC, non-clear cell RCC, and mixed type. Cut-off values of NLR ranged from 2.0 to 5.0. The HR and 95% CI data were extracted from the multivariate Cox regression analysis in 25 studies and univariate analysis in two studies. The mean age ranged from 56.3 to 69 years and the mean follow-ups ranged from 7.6 to 107.6 months. The NOS scores ranged from 6 to 8, showing a moderate to high quality of the included studies (Table S1).

**Table 1:** Characteristics of the studies included in the meta-analysis

Authors and year	Country	Sample size	Histology type	Stage	Mean age (years)	Treatment	Cut-off value and determined method		Outcome	Follow-up (Mean, months)	NOS score
Chen 2019 [18]	China	414	Clear	Non-metastatic	56.3	Radical or partial nephrectomy	2.17	Based on X-tile	OS, CSS	69.2	8
Huszno 2019 [19]	Poland	141	Clear	Metastatic	62	Tyrosine kinase inhibitors or IFN-α	3.68	ROC curve	OS, PFS	NA	7
Ishihara 2019 [20]	Japan	58	Mixed	Metastatic	NA	Nivolumab	3	Based on previous study	OS, PFS	13.1	7
Shirotake 2019 [11]	Japan	54	Clear	Metastatic	69	Nivolumab	2.89	Median value of NLR	PFS	10.6	7
Silagy 2019 [21]	USA	100	Non-clear	Metastatic	61	Cytoreductive nephrectomy	4.0	Median value of NLR	OS	13.3	7
Suzuki 2019 [22]	Japan	65	Mixed	Metastatic	68	Nivolumab	5	ROC curve	OS, PFS	9.5	6
Takagi 2019 [23]	Japan	71	Clear	Metastatic	66	Tyrosine Kinase Inhibitor Therapy	3	Mean value of NLR	OS	NA	7
Tu 2019 [24]	China	76	Papillary	Non-metastatic	57.5	Radical or partial nephrectomy	2.5	ROC curve	DFS	28.0	6
Widz 2019 [25]	Poland	196	Mixed	Non-metastatic	61	Radical or partial nephrectomy	2.69	ROC curve	OS	68	7
Zheng 2019 [26]	China	662	Mixed	Non-metastatic	61.7	Nephrectomy	3.2	Based on X-tile	OS, MFS	50.35	7
Kim 2018 [27]	Korea	190	Mixed	Metastatic	NA	Immunotherapy or/and VEGF	2.56	Median value of NLR	OS	107.6	7
Zahoor 2018 [28]	Austria	90	Clear	Metastatic	65	Nivolumab	4.2	NA	PFS	7.6	7
Zheng 2018 [29]	China	635	Mixed	Non-metastatic	61.71	Radical or partial nephrectomy	3.5	Based on X-tile	OS, CSS	48.40	7
Chang 2017 [30]	China	185	Clear	Localized	NA	Radiofrequency ablation	2.79	ROC curve	DFS, RFS	81	7
Dalpiaz 2017 [10]	USA	587	Clear	Non-metastatic	65	Radical or partial nephrectomy	2.0	Based on previous study	OS, CSS, MFS	NA	8
Jeyakumar 2017 [31]	USA	42	Mixed	Metastatic	61	VEGF and ICIs	3	Based on previous study	OS, PFS	NA	7
Grivas 2014 [32]	Greece	114	Mixed	Non-metastatic	64	Radical nephrectomy	2.7	Based on previous study	OS, DFS	69	7
Viers 2014 [33]	USA	827	Clear	Localized	65	Radical nephrectomy	4.0	NA	OS, DFS	111	7
Cetin 2013 [34]	Turkey	100	Mixed	Metastatic	58	IFN-α + VEGF	3.04	NA	OS, PFS	15	7
de Martino 2013 [35]	Austria	281	Non-clear	Localized	63	Radical or partial nephrectomy	3.6	ROC curve	DFS	37	7
Fox 2013 [36]	Australia	362	Mixed	Locally advanced/metastatic	62	lapatinib vs. antiangiogenic	3	Median value of NLR	OS	NA	7
Keizman 2013 [37]	Israel	244	Mixed	Metastatic	63	Sunitinib	3	Regression tree analysis	OS, PFS	55	6
Pichler 2013 [38]	Austria	678	Clear	Non-metastatic	NA	Radical or partial nephrectomy	3.3	Based on previous study	OS, CSS, MFS	44	7
Santoni 2013 [39]	Italy	97	Clear	Metastatic	64	Everolimus	3	Statistical method	OS, PFS	46.9	7
Ohno 2010 [40]	Japan	192	NA	Non-metastatic	60	Nephrectomy	2.7	Statistical method	RFS	93	7

*Abbreviations*: *OS* overall survival, *DFS* disease-free survival, *RFS* recurrence-free survival, *PFS* progression-free survival, *MFS* metastasis-free survival, *CSS* cancer-specific survival, *ICIs* immnue checkpoint inhibitors, *ROC* curve receiver operating characteristic curve, *NA* not available, *NOS* score Newcastle-Ottawa Scale score

### NLR and OS in RCC

**Fig. 2:**
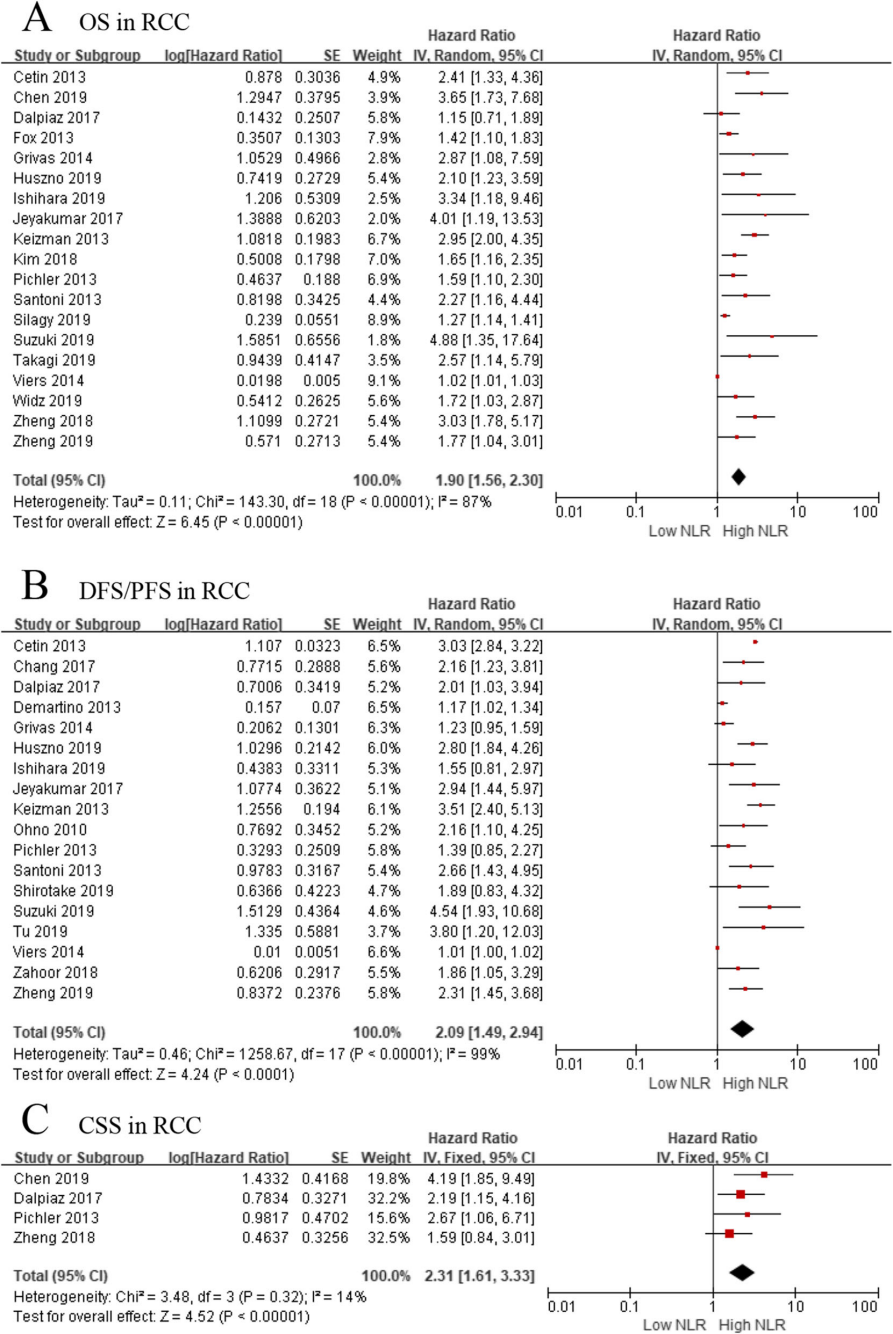
**a** Effect of the NLR on overall survival, **b** effect of the NLR on disease-free survival/progression-free survival, **c** effect of the NLR on cancer-specific survival

Nineteen studies, comprising 5768 patients, evaluated the association of NLR with OS in RCC patients. Because of significant heterogeneity (*I*^*2*^ = 87%, *p* < 0.001), a random-effect model was applied to investigate the prognostic role of NLR. The forest plot indicated that elevated pretreatment NLR was significantly associated with shorter OS in the overall population (pooled HR: 1.90, 95% CI: 1.56–2.30, *p* < 0.001, Fig. 2).

**Fig. 3:**
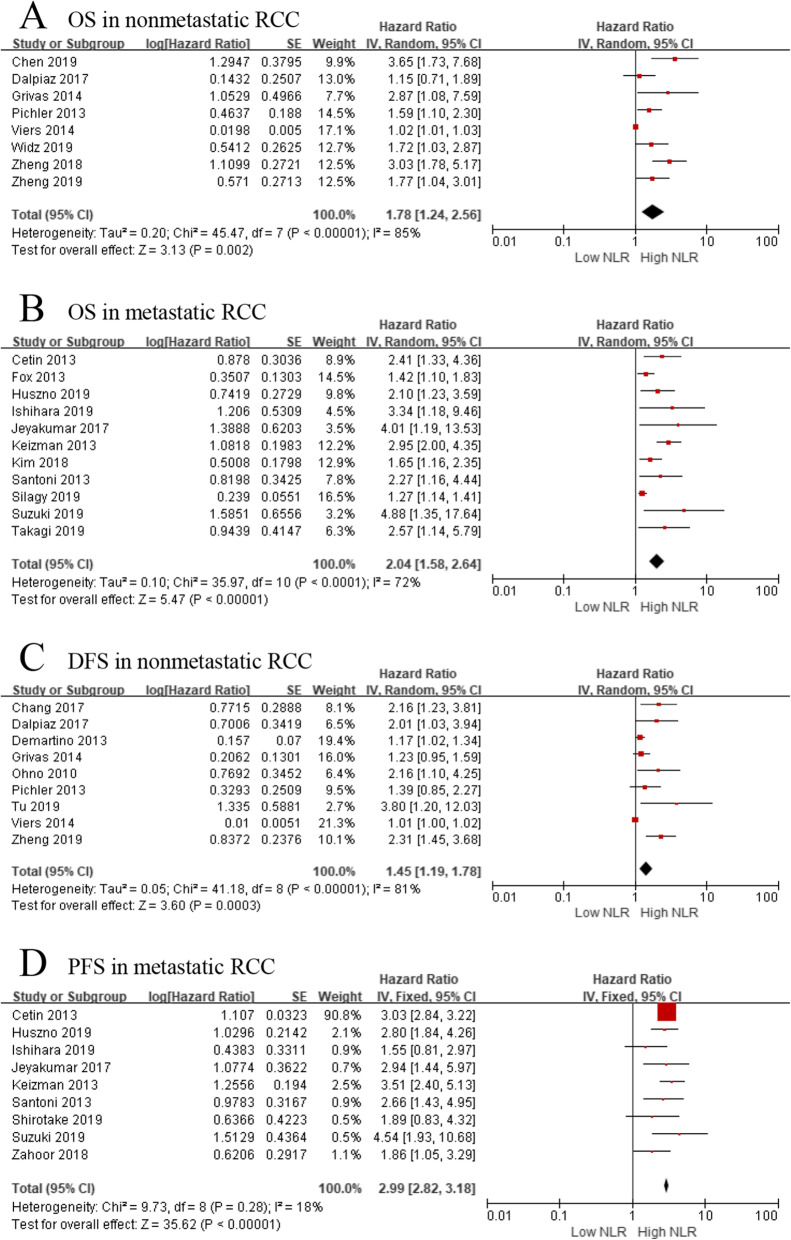
**a** Effect of the NLR on OS in nonmetastatic RCC, **b** effect of the NLR on OS in metastatic RCC, **c** effect of the NLR on DFS in nonmetastatic RCC, **d** effect of the NLR on PFS in metastatic RCC

When we evaluated the relationship between pretreatment NLR and OS in nonmetastatic (localized) RCC, we investigated 8 studies, including 4113 patients, while the same relationship was possible in 11 studies including 1420 patients with metastatic RCC. Meta-analysis showed that elevated NLR was significantly associated with worse OS in patients with nonmetastatic and metastatic RCC (pooled HR = 1.78, 95% CI: 1.24–2.56, *p* < 0.001; pooled HR = 2.04, 95% CI: 1.58–2.64, *p* < 0.001, respectively). Of note, heterogeneity was still obvious in nonmetastatic (*I*^*2*^ = 85%, *p* < 0.001) and metastatic populations (*I*^*2*^ = 72%, *p* < 0.001) (Fig. 3).

To explore whether a single study affected heterogeneity and conclusions, we performed a sensitivity analysis by sequentially excluding each single study in turn. After removing Viers’ study, heterogeneity among studies was reduced (*I*^*2*^ = 48%, *p* = 0.07) in nonmetastatic RCC. Similarly, after removing Silagy’s study, heterogeneity was reduced (*I*^*2*^ = 46%, *p* = 0.05) in metastatic RCC. Overall, the results of sensitivity analyses did not affect the conclusions described above and validated the robustness of our findings.

### NLR and DFS, PFS in RCC

When we investigated the association between NLR and DFS/PFS, 18 studies with 2735 patients were selected. The forest plot revealed that a high pretreatment NLR resulted in worse DFS/PFS in overall population (pooled HR = 2.09, 95% CI: 1.49–2.94, *p* < 0.001; *I*^*2*^ = 99%; Fig. 2).

When we further evaluated the relationship between NLR and DFS in nonmetastatic RCC patients, we included 9 studies with 3602 patients. The forest plot revealed that elevated NLR showed a significant association with worse DFS (pooled HR = 1.45, 95% CI: 1.19–1.78, *p* < 0.001; *I*^*2*^ = 81%; Fig. 3). As for the relationship between NLR and PFS in metastatic RCC patients, meta-analyses based on 9 studies indicated that high pretreatment NLR was significantly associated with poorer PFS (pooled HR = 2.99, 95% CI: 2.82–3.18, *p* < 0.001; *I*^*2*^ = 18%; Fig. 3).

Consequently, sensitivity analyses were also performed in nonmetastatic RCC patients. The results showed that the omission of any one study, except Viers’ study, did not significantly affect heterogeneity. However, heterogeneity was still relatively significant after the removal of Viers’ study. Since different study features were involved, we further performed subgroup analyses to explore the source of heterogeneity (Table 2). In the subgroup analysis based on sample sizes, elevated pretreatment NLR was significantly associated with poorer DFS in both sample sizes ≥200 (pooled HR = 1.57, 95% CI: 1.09–2.26, *p* = 0.02; *I*^*2*^ = 69%) and sample sizes < 200 (pooled HR = 1.85, 95% CI: 1.17–2.92, *p* = 0.008; *I*^*2*^ = 60%). Moreover, histology type, mean age, cut-off value of NLR, treatment strategy, the mean follow-up months, and other study features did not affect the relationship between NLR and DFS in nonmetastatic RCC patients. Interestingly, when stratified by race of patients, heterogeneity was significantly reduced and results demonstrated that elevated NLR was significantly associated with pooer DFS in both Asian and Caucasian RCC patients (pooled HR = 2.31; 95% CI: 1.70–3.14, *p* < 0.001; *I*^*2*^ = 0%; pooled HR = 1.21, 95% CI: 1.08–1.36, *p* = 0.001; *I*^*2*^ = 0%, respectively).

**Table 2:** Subgroup analysis for DFS in non-metastatic RCC patients

Subgroup	No. of studies	No. of patients	HR (95% CI)	*P* value	Heterogeneity	
					I^2^ (%)	Ph
Overall	9	3602	1.45 (1.19–1.78)	< 0.001	81	< 0.001
Study for subgroup analysis	8	2775	1.63 (1.27–2.07)	< 0.001	62	0.01
Race						
Caucasian	4	1660	1.21 (1.08–1.36)	0.001	0	0.43
Asian	4	1115	2.31 (1.70–3.14)	< 0.001	0	0.85
Sample size						
≥ 200	4	567	1.57 (1.09–2.26)	0.02	69	0.02
< 200	4	2208	1.85 (1.17–2.92)	0.008	60	0.06
Histology type						
Clear cell carcinoma	3	1450	1.75 (1.27–2.43)	< 0.001	0	0.46
Others	5	1325	1.58 (1.16–2.15)	0.004	71	0.009
Mean age (years)						
≥ 65	1	587	–	–	–	–
< 65	7	2188	1.59 (1.24–2.06)	< 0.001	65	0.009
Treatment						
Nephrectomy	7	2590	1.56 (1.22–2.00)	0.001	61	0.02
Radiofrequency ablation	1	185	–	–	–	–
Cut-off value of NLR						
≥ 2.75	4	1806	1.61 (1.10–2.35)	0.01	73	0.01
< 2.75	4	969	1.79 (1.14–2.81)	0.01	54	0.09
Mean time of follow-up (months)						
≥ 60	3	491	1.65 (1.07–2.55)	0.02	59	0.09
< 60	4	1697	1.66 (1.07–2.56)	0.02	73	0.01

### NLR and CSS in RCC

Four studies, comprising 2314 patients, provided data on the association of NLR with CSS. The forest plot indicated that higher pretreatment NLR was significantly associated with worse CSS (pooled HR = 2.31, 95% CI: 1.61–3.33, *p* < 0.001, *I*^*2*^ = 14%; Fig. 2). Considering that the patients enrolled in these four studies were nonmetastatic RCC, we did not further investigated the association between NLR and CSS in metastatic RCC patients. Furthermore, a sensitivity analysis was performed to explore whether a single study affected heterogeneity and conclusions. After removing Zheng’s study, heterogeneity among studies was markedly changed (*I*^*2*^ = 0%, *p* = 0.47) in nonmetastatic RCC. However, the pooled HR recalculated did not affect the conclusion described above, which validated the strength of our results.

### Subgroup analyses based on histology type

Considering that histology type may be the source of heterogeneity, we especially performed subgroup analyses based on histology types of RCC patients. The results revealed that histology types could change heterogeneity significantly. As shown in Table 3, pretreatment NLR could predict the outcome of clear cell RCC patients, including OS in nonmetastatic RCC (HR = 1.75, 95%CI: 1.03–2.99, *p* = 0.04; *I*^*2*^ = 69%), OS in metastatic RCC (HR = 2.24, 95%CI: 1.55–3.25, *p* < 0.001; *I*^*2*^ = 0%), DFS in nonmetastatic RCC (HR = 1.75, 95%CI: 1.27–2.43, *p* < 0.001; *I*^*2*^ = 0%), PFS in metastatic RCC (HR = 2.40, 95%CI: 1.82–3.18, p < 0.001; *I*^*2*^ = 0%), and CSS in nonmetastatic RCC (HR = 2.77, 95%CI: 1.78–4.32, p < 0.001; *I*^*2*^ = 0%).

**Table 3:** Subgroup analyses based on histology type

Subgroup	No. of studies	No. of patients	HR (95% CI)	*P* value	Heterogeneity	
					I^2^ (%)	Ph
OS in nonmetastatic RCC						
Overall	8	4113	1.78 (1.24–2.56)	< 0.001	85	0.002
Clear cell RCC	4	2506	1.75 (1.03–2.99)	0.04	69	0.04
Others	4	1607	2.15 (1.61–2.87)	< 0.001	6	0.36
OS in metastatic RCC						
Overall	11	1420	2.04 (1.58–2.64)	< 0.001	72	< 0.001
Clear cell carcinoma	3	309	2.24 (1.55–3.25)	< 0.001	0	0.92
Others	8	1111	1.99 (1.47–2.68)	< 0.001	77	< 0.001
DFS in nonmetastatic RCC						
Overall	8	3602	1.45 (1.19–1.78)	< 0.001	81	< 0.001
Clear cell carcinoma	3	1450	1.75 (1.27–2.43)	< 0.001	0	0.46
Others	5	1325	1.58 (1.16–2.15)	0.004	71	0.009
PFS in metastatic RCC						
Overall	9	891	2.99 (2.82–3.18)	< 0.001	18	0.28
Clear cell carcinoma	4	382	2.40 (1.82–3.18)	< 0.001	0	0.64
Others	5	509	3.02 (2.84–3.22)	< 0.001	28	0.24
CSS in nonmetastatic RCC						
Overall	4	2314	2.31 (1.61–3.33)	< 0.001	14	0.32
Clear cell carcinoma	3	1679	2.77 (1.78–4.32)	< 0.001	0	0.47
Others	1	635	–	–	–	–

### OS and PFS in patients treated with ICIs

**Fig. 4:**
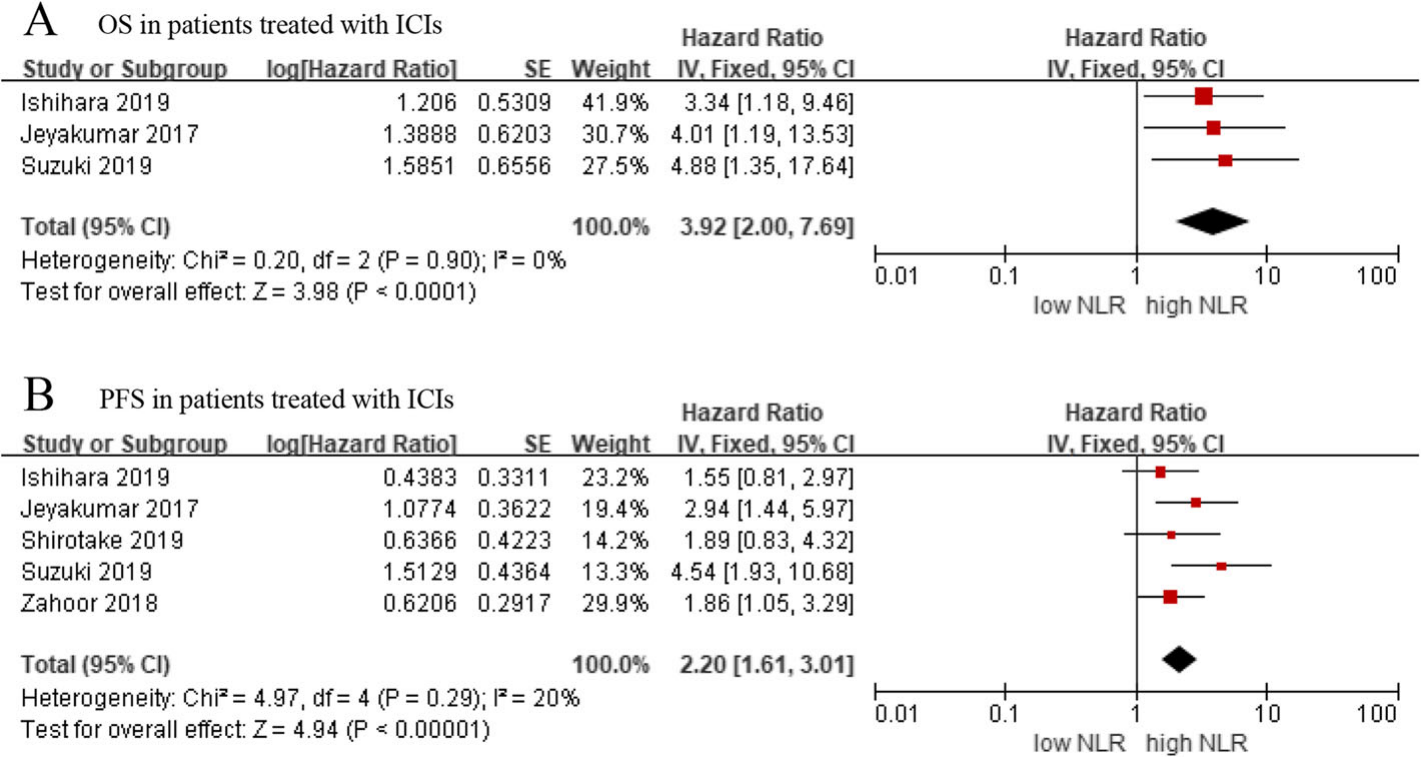
**a** OS in RCC patients treated with ICIs, **b** PFS in RCC patients treated with ICIs

In addition to the above analysis, we also investigated the prognostic role of NLR in metastatic RCC patients treated with immune checkpoint inhibitors (ICIs). The results indicated that the high level of NLR was significantly associated with worse OS (HR = 3.92, 95% CI: 2.00–7.69, *p* < 0.001; *I*^*2*^ = 0%) and PFS (HR = 2.20, 95% CI: 95% CI: 1.61–3.01, p < 0.001; *I*^*2*^ = 20%) (Fig. 4).

### Publication bias

Publication bias was assessed, respectively for OS, DFS/PFS, and CSS without considering the stage of RCC patients. For both OS and DFS/PFS, the funnel plots were asymmetric (Fig. S1). Disappointingly, the Egger’s test also showed some degree of publication bias (both *p* < 0.001). Therefore, the trim and fill method was carried out to examine the funnel plot’s asymmetry by using hypothetically non-published studies. The recalculated results indicated that elevated NLR was significantly associated with OS (HR: 1.51, 95%CI: 1.28–1.79, *p* < 0.001) and DFS/PFS (HR: 1.96, 95%CI: 1.42–2.72, *p* < 0.001), indicating the stability of the results (Fig. S2). For CSS, the funnel plot was relatively symmetric (Fig. S1). Moreover, the Egger’s test showed that there was no obvious publication bias (*p* = 0.285).

### Quality of evidence

The assessment of the quality of evidence was performed for OS, DFS/PFS, and CSS which were critical in evaluating the prognosis of RCC patients. The results showed that the quality of evidence of OS and DFS/PFS was both “very low” because of observational studies, some degree of publication bias, and significant heterogeneity. However, the quality of evidence of CSS was “low” due to observational studies (Table S2).

## Discussion

Prognostic markers have multiple applications in the diagnosis, treatment, and prediction of clinical outcome and can contribute to choosing the best treatment strategy. Pretreatment NLR, as a prognostic predictor for RCC, has been evaluated by numerous studies, but they reported controversial results. Thus, the aim of this systematic review and meta-analysis of 6461 RCC patients was to clarify the prognostic value of pretreatment NLR in RCC patients. The results indicated that elevated pretreatment NLR was significantly associated with poorer OS, DFS, and CSS in nonmetastatic RCC patients. Similarly, high pretreatment NLR also showed significant association with worse OS and PFS in metastatic RCC patients. To further explore the source of heterogeneity, we performed subgroup analyses according to the features of enrolled studies, which also demonstrated the stability and reliability of our results. Collectively, the pooled data from the present systematic review and meta-analysis demonstrated that NLR may serve as a prognostic indicator in RCC patients and would be helpful in guiding clinical decision-making and selecting individualized treatment strategies.

It is largely recognized that systemic inflammatory response and tumor microenvironment are essential in the development and progression of cancer [5, 41]. Some researchers have shown cancer-related inflammatory response consists of cytokines, chemokines, transcription factors, and inflammatory cells, which play decisive roles at different stages of tumor development including initiation, promotion, malignant conversion, invasion, and metastasis [42, 43]. Hence, several inflammatory biomarkers, such as platelet-to-lymphocyte ratio (PLR) [44], lymphocyte-to-monocyte ratio (LMR) [45], C-reavtive protein to albumin ratio (CAR) [46], C-reactive protein (CRP) [47] and modified Glasgow Prognostic Score (mGPS) [48] are the potential prognostic biomarkers in RCC patients.

Recently, various studies investigated the prognostic value of NLR in RCC patients and the function of neutrophils and lymphocytes may be responsible for the underlying mechanisms. Neutrophils are usually regarded as an important part in the acute phase of inflammation and confer resistance against microbes. Some studies showed that neutrophils were involved in cancer development. Neutrophils could directly affect tumor cells to promote cancer progression. Moreover, neutrophils may indirectly change the tumor microenvironment to promote cancer metastasis [49]. In addition, neutrophils could release tumor growth promoters or immunoregulatory mediators, including vascular endothelial growth factor (VEGF) to affect cancer progression, which is generally regarded as an important part in tumor angiogenesis and has been indicated by the inhibitory effects of anti-VEGF antibodies on tumor growth in vivo [50–52]. By contrast, lymphocytes reflect on cell-mediated immunity and are essential in anti-tumor immune responses. Increased infiltration of lymphocytes in the tumor region has been associated with better responsiveness to therapy and better prognosis in patients with solid tumors [53]. Moreover, lymphopenia, with a decrease in CD4 + T-cells, which are often observed in cancer patients, compromises the anti-tumor response mediated by lymphocytes [54]. In other words, NLR not only reflects the inflammatory response in patients, but it also represents the decline of anti-tumor immunity, thus bringing a new perspective in determining the outcome of RCC patients.

Several meta-analyses have discussed the relationship between NLR and prognosis in RCC patients [55, 56]. Their results were similar to our results, but these meta-analyses did not perform sensitivity analysis and subgroup analysis to evaluate the source of heterogeneity and assess the publication bias. Furthermore, the advent of ICIs has changed the management of metastatic RCC. In particular, we investigated OS and PFS in metastatic RCC patients treated with ICIs, and the negative association was also confirmed in these RCC patients. Additionally, in this analysis, we found that heterogeneity was significantly reduced by dividing the study population according to the race of patients, namely, Caucasian and Asian RCC patients. Besides, we also noted that heterogeneity was significantly reduced by limiting the histology type to clear cell RCC. Therefore, when we explore the clinical role of NLR, it would be better to understand the histology type of patients. Moreover, there were several different defined methods about NLR in our studies. Some studies regarded NLR as a continuous variable, whereas some studies divided it into two groups based on ROC curve, median value, X-tile, and other methods. These different defined methods could potentially lead to heterogeneity. Of note, our study mainly evaluated the prognostic value of the pretreatment NLR. Several studies have shown that the post-treatment NLR was also an effective prognostic biomarker in RCC patients [44, 57, 58]. Therefore, the dynamic detection of the peripheral blood NLR level during treatment could play a more important prognostic role for RCC patients.

This study has several limitations that should be acknowledged. First, most of the included studies were retrospective and some of these studies had small sample sizes. Second, heterogeneity in some subgroup analyses was moderate or high. The possibility of selection biases or other unidentified confounders could not be completely avoided. Third, a certain degree of publication bias in our study may weaken the quality of evidence. Thus, the trim and fill method was performed and the adjusted results validated the stability of our results. Finally, there was no established cut-off value of NLR. Most scholars selected a cut-off value based on the highest sensitivity and specificity or used predefined cut-off values from other studies. Therefore, more large-scale prospective studies are needed to establish the standard cut-off value of NLR and provide more evidence.

## Conclusions

Our meta-analysis demonstrated that elevated pretreatment NLR is an indicator associated with poor prognosis in RCC patients. As a potential prognostic biomarker, urologists could combine NLR with TNM stage, Fuhrman nuclear grade, histological subtype and other widely accepted prognostic indicators to more precisely predict the outcome of RCC patients.

## Supplementary information

**Additional file 1: Figure S1.** (a) Funnel plot of NLR and OS, (b) funnel plot of NLR and DFS/PFS, (c) funnel plot of NLR and CSS

**Additional file 2: Figure S2.** (a) Funnel plot adjusted with trim and fill method for OS, (b) for DFS/PFS.

**Additional file 3: Table S1.** Newcastle-Ottawa scale score of the reviewed studies

**Additional file 4:Table S2.** Evaluation of the quality of evidence according to GRADE system

## Data Availability

All data generated or analysed during this study are included in this published article.

## Abbreviations

CAR: C-reactive protein to albumin ratio
CI: Confidence intervals
CRP: C-reactive protein
CSS: Cancer-specific survival
DFS: Disease-free survival
GRADE: Grading of Recommendations Assessment, Development, and Evaluation
HR: Hazard ratio
ICIs: Immune checkpoint inhibitors
LMR: Lymphocyte-to-monocyte ratio
mGPS: Modified Glasgow Prognostic Score
NLR: Neutrophil-to-lymphocyte ratio
OS: Overall survival
PLR: Platelet-to-lymphocyte ratio
PFS: Progression-free survival
RCC: Renal cell carcinoma
ROC curve: Receiver operating characteristic curve
