# Supplementary figures and images for: Prognostic value of pretreatment neutrophil-to-lymphocyte ratio in renal cell carcinoma: a systematic review and meta-analysis
Source: BMC Urol. 2020 Jul 6;20:90. doi: 10.1186/s12894-020-00665-8 (PMC7339475; doi:10.1186/s12894-020-00665-8)

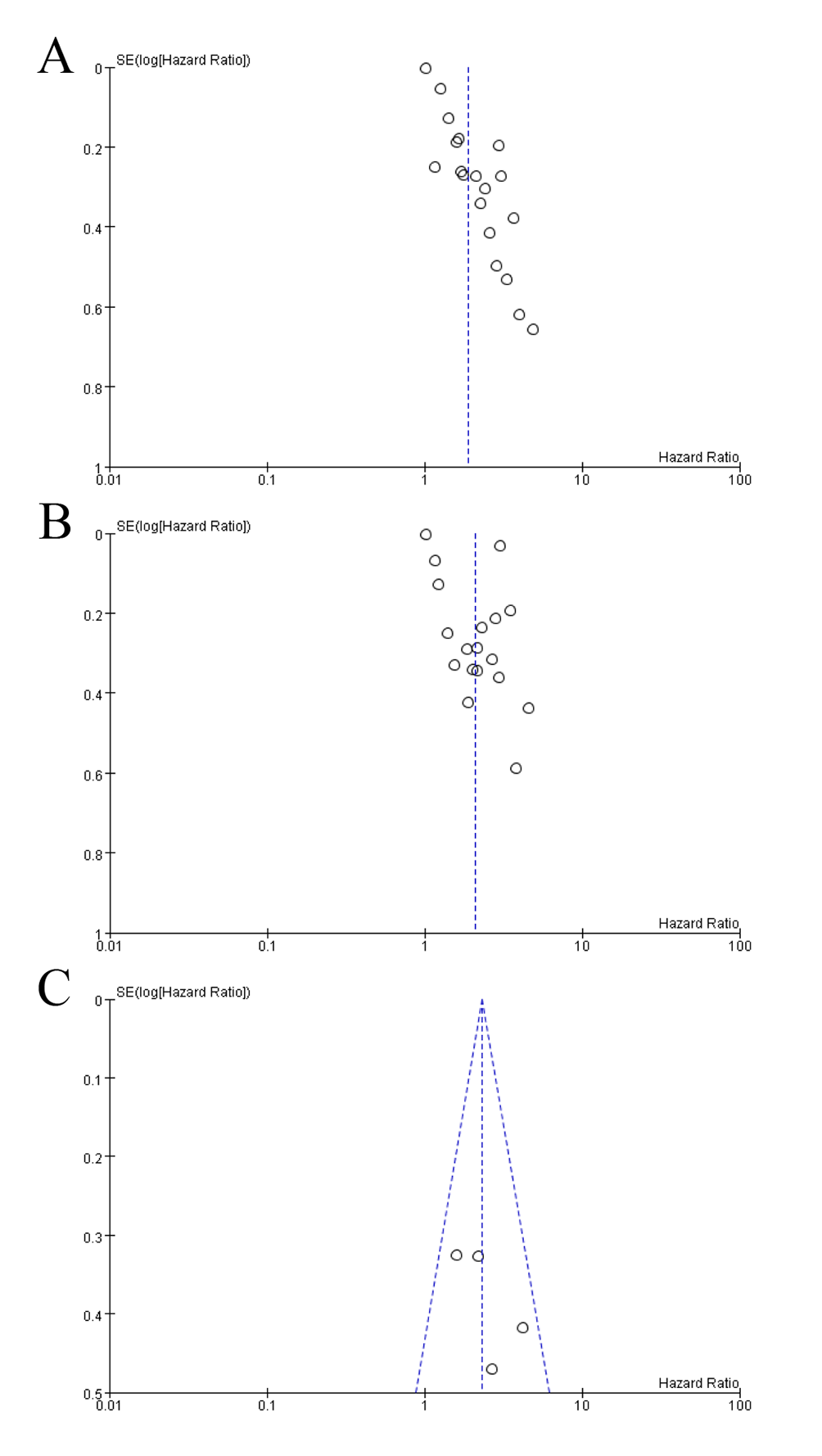

Supplement: Supplementary file 1 — Additional file 1: Figure S1. (a) Funnel plot of NLR and OS, (b) funnel plot of NLR and DFS/PFS, (c) funnel plot of NLR and CSS [file 12894_2020_665_MOESM1_ESM.tif]

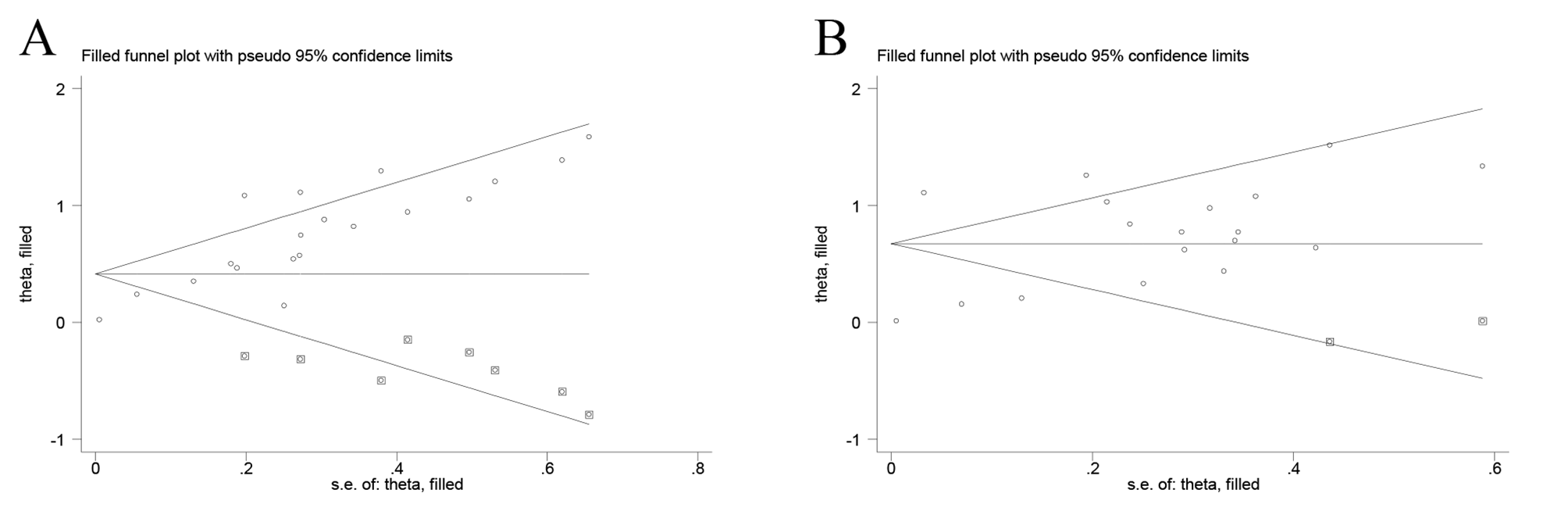

Supplement: Supplementary file 2 — Additional file 2: Figure S2. (a) Funnel plot adjusted with trim and fill method for OS, (b) for DFS/PFS. [file 12894_2020_665_MOESM2_ESM.tif]
