# Supplementary material for: Prognostic value of pretreatment neutrophil-to-lymphocyte ratio in renal cell carcinoma: a systematic review and meta-analysis
Source: BMC Urol. 2020 Jul 6;20:90. doi: 10.1186/s12894-020-00665-8 (PMC7339475; doi:10.1186/s12894-020-00665-8)
Supplement: Supplementary file 3 — Additional file 3: Table S1. Newcastle-Ottawa scale score of the reviewed studies [file 12894_2020_665_MOESM3_ESM.docx]

| Table S1 Newcastle-Ottawa scale score of the reviewed studies | | | | | | | | | |
| --- | --- | --- | --- | --- | --- | --- | --- | --- | --- |
| Study | Selection (4 stars) | | | | Comparability  (2 stars) | Outcome (3 stars) | | | Total  score |
|  | Representativeness of the exposed cohort | Selection of the non exposed cohort | Ascertainment  of exposure | Demonstration that outcome of interest was not present at start of study |  | Assessment of outcome | Was follow up long enough for outcomes to occur? | Adequacy of follow up of cohort |  |
| Chen | ★ | ★ | ★ | ★ | ★ | ★ | ★ | ★ | 8 |
| Huszno | ★ | ★ | ★ | ★ | - | ★ | ★ | ★ | 7 |
| Ishihara | ★ | ★ | ★ | ★ | - | ★ | ★ | ★ | 7 |
| Shirotake | ★ | ★ | ★ | ★ | - | ★ | ★ | ★ | 7 |
| Silagy | ★ | ★ | ★ | ★ | - | ★ | ★ | ★ | 7 |
| Suzuki | - | ★ | ★ | ★ | - | ★ | ★ | ★ | 6 |
| Takagi | ★ | ★ | ★ | ★ | - | ★ | ★ | ★ | 7 |
| Tu | ★ | ★ | - | ★ | - | ★ | ★ | ★ | 6 |
| Widz | ★ | ★ | ★ | ★ | - | ★ | ★ | ★ | 7 |
| Zheng | ★ | ★ | ★ | ★ | - | ★ | ★ | ★ | 7 |
| Kim | ★ | ★ | ★ | ★ | - | ★ | ★ | ★ | 7 |
| Zahoor | ★ | ★ | ★ | ★ | - | ★ | ★ | ★ | 7 |
| Zheng | ★ | ★ | ★ | ★ | - | ★ | ★ | ★ | 7 |
| Chang | ★ | ★ | ★ | ★ | - | ★ | ★ | ★ | 7 |
| Dalpiaz | ★ | ★ | ★ | ★ | ★ | ★ | ★ | ★ | 8 |
| Jeyakumar | ★ | ★ | ★ | ★ | - | ★ | ★ | ★ | 7 |
| Grives | ★ | ★ | ★ | ★ | - | ★ | ★ | ★ | 7 |
| Viers | ★ | ★ | ★ | ★ | - | ★ | ★ | ★ | 7 |
| Cetin | ★ | ★ | ★ | ★ | - | ★ | ★ | ★ | 7 |
| de Martino | ★ | ★ | ★ | ★ | - | ★ | ★ | ★ | 7 |
| Fox | ★ | ★ | ★ | ★ | - | ★ | ★ | ★ | 7 |
| Keizman | ★ | ★ | ★ | ★ | - | - | ★ | ★ | 6 |
| Pichler | ★ | ★ | ★ | ★ | - | ★ | ★ | ★ | 7 |
| Santoni | ★ | ★ | ★ | ★ | - | ★ | ★ | ★ | 7 |
| Ohno | ★ | ★ | ★ | ★ | - | ★ | ★ | ★ | 7 |
| -: The data were not available in this study | | | | | | | | | |
