# Supplementary material for: Prognostic value of pretreatment neutrophil-to-lymphocyte ratio in renal cell carcinoma: a systematic review and meta-analysis
Source: BMC Urol. 2020 Jul 6;20:90. doi: 10.1186/s12894-020-00665-8 (PMC7339475; doi:10.1186/s12894-020-00665-8)
Supplement: Supplementary file 4 — Additional file 4:Table S2. Evaluation of the quality of evidence according to GRADE system [file 12894_2020_665_MOESM4_ESM.docx]

| **Table S2** Evaluation of the quality of evidence according to GRADE system | | | | | | | | | | |
| --- | --- | --- | --- | --- | --- | --- | --- | --- | --- | --- |
| Quality assessment | | | | | | | No. of patients | Hazard Ratios (95% CI) | Quality | Importance |
| No. of studies | Design | Risk of bias | Inconsistency | Indirectness | Imprecision | Other considerations |  |  |  |  |
| OS |  |  |  |  |  |  |  |  |  |  |
| 19 | observational studies | no serious risk of bias | serious^1^ | no serious  indirectness | no serious imprecision | reporting bias^2^ | 5768 | 1.90 (1.56-2.30) | Very low | Critical |
| DFS/PFS |  |  |  |  |  |  |  |  |  |  |
| 18 | observational studies | no serious risk of bias | serious^1^ | no serious  indirectness | no serious imprecision | reporting bias^2^ | 4493 | 2.09 (1.49-2.94) | Very low | Critical |
| CSS |  |  |  |  |  |  |  |  |  |  |
| 4 | observational studies | no serious risk of bias | no serious  inconsistency | no serious  indirectness | no serious imprecision | none | 2314 | 2.31 (1.61-3.33) | Low | Critical |
| ^1^The heterogeneity of this outcome was obvious between studies.  ^2^The shape of funnel plot was not symmetric. The Egger’s test was further performed. The result indicated significant publication bias for studies, with merged OS (*p*<0.01).  ^3^The shape of funnel plot was not symmetric. The Egger’s tests was further performed. The result indicated significant publication bias for studies, with merged DFS/PFS (*p*<0.01). | | | | | | | | | | |
